# Cakes fortified with papaya seeds effectively protects against CCl4-induced immunotoxicity

**DOI:** 10.1007/s11356-023-30172-w

**Published:** 2023-10-10

**Authors:** Hanaa S. S. Gazwi, Osama I. A. Soltan, Sanaa M. Abdel-Hameed

**Affiliations:** 1 Department of Agricultural Chemistry, Faculty of Agriculture, Minia University, El-Minia, 61519 Egypt; 2 Department of Food Science, Faculty of Agriculture, Minia University, El-Minia, 61519 Egypt

**Keywords:** Cakes enhanced with papaya seeds, CCl4, Histopathology, Herbal remedies, Oxidative stress, Antioxidant

## Abstract

Maintaining a robust immune system and safeguarding the liver from toxins are crucial for overall health. The study aimed to investigate the immunostimulant effects of papaya seed-enriched cakes (CPS) in countering carbon tetrachloride (CCl4)-induced immunocytotoxicity in rats (*n* = 48). The rats were divided into six groups (8 each): a control group (Group 1), rats fed cakes containing 15% papaya seeds (Group 2 — CPS), rats exposed only to CCl4 (Group 3 — CCl4), rats injected with CCl4 and administered silymarin (Group 4 — CCl4 + S), rats receiving both CCl4 and cakes with papaya seeds (Group 5 — CCl4 + CPS), and rats receiving both CCl4 and silymarin with papaya seed-enriched cakes (Group 6 — CCl4 + CPS + S). HPLC analysis of papaya seeds revealed the presence of ten polyphenol compounds, with quercetin, apigenin, and catechin identified as major flavonoids, along with pyrogallol, ellagic, and gallic acid as predominant phenolic acids. These compounds displayed potent antioxidant activity, attributed to the seeds’ high total phenolic and flavonoid content. The administration of CCl4 significantly affected hematological parameters, liver enzymes, hepatic oxidative stress, levels of TNF-α, IL-6, IgG, as well as IgM. However, rats fed with CPS exhibited mitigation of CCl4-induced toxic effects on hematological parameters and hepatotoxicity. CPS consumption enhanced the antioxidant system, improved inflammatory markers, and immune parameters, restoring them to normal levels. Histopathological analysis confirmed CPS’s ability to reduce CCl4-induced hepatocellular necrosis. Immunohistochemical assessment further revealed reduced immunoreactivity against cleaved caspase-3 expression and increased COX2 immunoreactivity, indicating hepatocellular regeneration in CPS. The combination of CPS and silymarin demonstrated even more notable improvements, suggesting augmented protective impacts against CCl4-induced immunosuppression and hepatotoxicity. In conclusion, CPS exhibited antioxidant properties and effectively protected against CCl4-induced immunotoxicity and hepatotoxicity, with additional benefits observed when combined with silymarin. These findings emphasize the potential health advantages of incorporating papaya seeds into food products, promoting immune system health, and safeguarding against liver damage induced by hazardous agents like CCl4.

## Introduction

Food consumption plays a critical role in preventing hunger and maintaining human health (Kulczyński et al. 2020). The growing emphasis on health has led to an increasing demand for functional foods, as consumers recognize the significant link between food and well-being. They seek foods that not only satisfy hunger and provide essential nutrients but also help safeguard against nutritional diseases and promote overall health (Goetzke et al. 2014).

Bakery products, particularly cakes, are widely consumed worldwide, favored by both younger and elderly populations in both urban and rural areas (Hosseini et al. 2018). Cakes are typically composed of ingredients such as flour, fat, sweeteners, and liquids. However, due to their high sugar and fat content, cakes contribute significantly to caloric intake (Al-Tamim 2014).

Food fortification involves enriching food with specific nutrients like proteins, minerals, and vitamins from animal or plant sources (Shehata and El-sayed 2020). These fortified foods are particularly beneficial for individuals with malnutrition or specific health conditions (Abril et al. 2012). Moreover, there has been a global interest in exploring the use of dietary plants and herbal remedies as alternatives to traditional medicine for preventing and treating diabetes mellitus (Hunt et al. 2000).

Papaya (*Carica papaya* L.), an indigenous tropical fruit of the Americas, has been cultivated in diverse geographical locations worldwide (FAO 2019). The processing of papaya into various products generates substantial agro-industrial waste, including peels and seeds, which make up about 50% of the fruit’s weight, with seeds accounting for an average of 14% (Venturini et al. 2012). To minimize waste, papaya seeds can be utilized since they contain bioactive compounds with potential benefits for the food industry. For instance, Piovesan et al. (2017) found that incorporating papaya seed extract in chicken sausage reduced lipid oxidation compared to the control group. Additionally, papaya seed extract showed potential in reducing oxidation in vegetable oils and foods (Sofi et al. 2016).

The immune system encompasses molecules, cells, and tissues that play a critical role in protecting animals from external invaders. This essential defense system consists of lymphoid organs, barriers, leukocytes, and proteins, such as antibodies and complement components (Blach-Olszewska and Leszek 2007). The liver contributes significantly to immune homeostasis through various mechanisms. Firstly, it serves as a central player in the immune response, safeguarding against blood-borne pathogens by virtue of its dual blood supply, ultimately preventing the widespread dissemination of nutritional antigens and microbes from the gut (Albillos et al. 2014). Secondly, it maintains immune response homeostasis by facilitating the production of essential compounds required for an effective immune response (Racanelli and Rehermann 2006). The liver carries out its role in monitoring anti-microbial activities through a coordinated effort involving antigen-presenting cells (APCs) and lymphocytes, which specifically surveil for both common and gut-derived pathogens. These APCs include Kupffer cells, sinusoidal endothelial cells from the liver, and dendritic cells (Gregory et al. 2002). Moreover, the liver houses T and B cells among its lymphocyte population, contributing to the adaptive immune response in both parenchyma and portal tracts. Additionally, the liver is rich in NK cells and unconventional lymphocytes that participate in its innate immune responses (Schildberg et al. 2008). Key immune system organs include the bone marrow, lymph nodes, appendix, spleen, and thymus (Blach-Olszewska and Leszek 2007). The spleen, a major secondary lymphoid organ, fulfills various immunological functions, including hematopoiesis and red blood cell clearance. Its unique structure allows for the filtration of pathogens and abnormal cells from the bloodstream and facilitates interactions between APCs and lymphocytes. Spleen-specific APCs play a pivotal role in regulating T and B cell responses to blood-borne antigens (Lewis et al. 2019). Lymph nodes, categorized as lymphoid organs, consist of lymphocytes within a delicate reticular stroma. They serve as filters for tissues and function as sites for lymphocyte development and origin to support normal physiological functions (Elmore and Bouknight 2017).

Carbon tetrachloride (CCl4) is a commonly used chlorinated hydrocarbon in various industries as a solvent and in medicine for treating hookworm disease (Rajat et al. 2007). CCl4 exposure can cause damage to various organs. While in most animal species, the liver is the primary organ affected by CCl4-induced toxicity, there is evidence suggesting an impact on the immune system as well (Guo et al. 2000). When metabolized by cytochrome P450, CCl4 forms the highly reactive trichloromethyl (CCl3) radical, which generates an abundance of oxygen radicals and phospholipid peroxides, ultimately initiating lipid peroxidation (Sreedevi et al. 2006). Exposure to CCl4 activates immune cells, leading to their infiltration into the damaged liver and subsequent release of inflammatory mediators, such as interleukins, COX-2, cytokines/chemokines, in response to oxidative stress (Ferguson et al. 2004). Prolonged administration of CCl4 results in an increased concentration of reactive oxygen species (ROS), leading to cirrhosis, fibrosis, and potentially hepatic carcinoma (Nakamura et al. 2000). While our bodies are capable of producing antioxidant enzymes, the intake of dietary antioxidants becomes crucial to bolster immunity and provide protection against the harmful impacts of oxidative stress and free radicals.

The present study aims to investigate the protective impacts of a cake fortified with papaya seeds or a combination of papaya seeds and silymarin against CCl4-induced immunomodulation in rats.

## Materials and methods

### Preparation of papaya seed

The papaya fruits underwent a meticulous washing process, and the seeds were carefully hand-collected for subsequent use. The collected seeds were thoroughly washed with water and then air-dried under shade at room temperature (25 °C), following the procedure outlined by Abdel-Hameed et al. (2023). After complete drying, the seeds were finely ground using a laboratory milling machine and passed through a 60-mesh screen to obtain a homogeneous seed flour. The resulting whole seed flour was then carefully packed in air-tight polyethylene bags and stored at a temperature of −18 °C until further utilization.

### Bioactive components of papaya seeds

To assess the bioactive components, 100 g of finely powdered papaya seeds was combined with 1000 mL of ethanol and then subjected to stirring using a magnetic stirrer for a duration of 3 h. Following stirring, the mixture underwent filtration using filter paper, and the resultant filtrate was subjected to vacuum evaporation by an evaporator. Subsequently, the obtained extract was employed for analysis (Gazwi 2020).

To assess the total phenolic content of the papaya seed powder, a calorimetric method utilizing the Folin-Ciocalteu reagent was employed, following the procedure outlined by Singleton et al. (1999). The phenolic content was quantified and expressed as milligrams of gallic acid equivalent (mg GAE) per gram of the sample.

For the determination of the total flavonoid content in the papaya seed powder, the method described by Zhishen et al. (1999) was adopted. The total flavonoid content was quantified and expressed as milligrams of quercetin equivalents (mg QE) per gram of the sample.

To assess the in vitro antioxidant activity of the papaya seed powder, the 1,1-diphenyl-2-picryl-hydrazyl (DPPH) assay was conducted, following the standard procedure described by Zhu et al. (2006).

### HPLC analysis for powdered papaya seeds

The HPLC system used for separation was an Agilent 1260 Infinity HPLC (Agilent, USA), consisting of a quaternary pump and a Kinetex® 5 μm EVO C18 100 mm × 4.6 mm column (Phenomenex, Santa Clara, CA, USA), with the operating temperature set at 30 °C. For the separation process, a ternary linear elution gradient was applied, using HPLC-grade water and 0.2% H_3_PO_4_ (v/v) (A), methanol (B), and acetonitrile (C). The detection of phenols and flavonoids was accomplished using a variable-wavelength detector set at 284 nm, following the established protocol by Soltan et al. (2023).

### Preparation of cakes

The control and supplemented chocolate cupcakes were prepared following the recipe outlined in the research published by Abdel-Hameed et al. (2023), who successfully demonstrated the use of papaya seeds as nutritive ingredients and antimicrobial agents in conjunction with wheat flour. This resulted in delectable and healthful chocolate cupcakes. The supplementation of cupcakes, up to a 15% level of papaya seeds, exhibited improved quality attributes and increased nutritional value, including higher protein content (16.89%), fiber content (3.28%), and ash content (4.17%). Among the supplemented cupcakes, those fortified with 15% papaya seeds showed the most promising results in preliminary tests and were consequently chosen for use in this study.

### Experimental animals

A total of 48 adult albino rats, aged around thirty, and weighing approximately 180 ± 5 g, were obtained from the Animal House at Nahda University’s Faculty of Pharmacy in Egypt. These rats were individually placed in cages and kept at a constant temperature of 25 ± 2 °C, with a 12-h light/dark cycle. They were provided with unrestricted access to both food and water, with the food being given ad libitum. Prior to the commencement of the experiment, the rats were given 1 week to adapt to their new surroundings. The basal diet used in the study was prepared using the approach outlined by Reeves et al. (1993). Ethical considerations were strictly followed throughout the experiment, in accordance with the regulations established by the Ethics Committee for the care and usage of animals, microorganisms, and living cell cultures in education and scientific research at the Faculty of Agriculture, Minia University (Approval No. MU/FA/009/12/22).

The experimental design involved six groups of rats, with eight rats in each group. The groups were as follows:

1. Group 1 (Control): normal rats fed on the basal diet only.
2. Group 2 (CPS): rats fed on a basal diet with 10% chocolate cupcakes which contained 15% of papaya seeds (Elsawy 2020) for 30 days.
3. Group 3 (CCl4): rats received CCl4 (1 ml/kg, 1:1 mixture with paraffin oil, intraperitoneally (IP), (Prabhu et al. 2010) twice weekly for 4 weeks, and fed on a basal diet only for 30 days.
4. Group 4 (CCl4 + S): rats received CCl4 (IP 1 ml/kg b.wt) and were given daily silymarin at 50 mg/kg/day orally (Nema et al. 2011) for 4 weeks and fed on a basal diet for 30 days. CCl4 was injected 30 min after oral treatment with silymarin.
5. Group 5 (CCl4 + CPS): rats received CCl4 (IP 1 ml/kg b.wt) and fed on a basal diet with 10% cupcakes which contained 15% of papaya seeds for 30 days.
6. Group 6 (CCl4 + CPS + S): rats received CCl4 (IP 1 ml/kg b.wt), silymarin (50 mg/kg b.wt/day) and fed on a basal diet with 10% cupcakes that contained 15% of papaya seeds.

After the end of experiment period, the rats were fasted overnight and euthanized under general anesthesia using IP injection of ketamine at dose 80 and xylazine at dose 10 mg/kg (Tsukamoto et al. 2018), following the method described by Farghadani et al. (2019). Blood samples were collected from the retro-orbital plexus of overnight fasting rats. Blood samples were divided, with some collected in EDTA-containing tubes, while the rest were placed in regular test tubes. The serum was then separated from the blood cells by subjecting the samples to centrifugation at 2500 rpm for 10 min. The separated serum samples were stored at a temperature of −20 °C; the serum was stored at −20 °C until further analysis. The animals were euthanized by cervical dislocation, and their livers were collected, weighed, washed with a physiological saline solution, and blotted dry on filter paper.

### Liver homogenate preparation

A portion of the liver tissue was homogenized in cold phosphate buffer saline (PBS, 0.1 mol and pH = 7.4) and then centrifuged at 4500× g for 20 min at 4 °C to obtain the supernatant. The resulting supernatants were collected for the evaluation of oxidative stress/antioxidant biomarkers.

### Hematological assessment

Hematological evaluation was performed using a hematology analyzer (CBC Mindray BC-3000 Plus) to measure various parameters, including red blood cell (RBC) count, white blood cell (WBC) count, hematocrit (HCT), hemoglobin (Hb) level, mean corpuscular hemoglobin (MCH), mean corpuscular volume (MCV), mean corpuscular hemoglobin concentration (MCHC), as well as the proportions of neutrophils, monocytes, lymphocytes, and blood eosinophils (Oudatzis et al. 2020).

### Biochemical analysis

For the biochemical analysis, levels of alanine aminotransferase (ALT), total protein, albumin, aspartate aminotransferase (AST), and alkaline phosphatase (ALP) were measured in serum using commercially available kits from Bio Diagnostic, Cairo, Egypt.

### Antioxidant/oxidative stress markers

The hepatic antioxidant/oxidative stress markers, including nitric oxide (NO), superoxide dismutase (SOD), reduced glutathione (GSH) activity, and malondialdehyde (MDA) as a marker of lipid peroxidation (LPO) levels were measured according to the manufacturer’s instructions.

### Immunoglobulin and inflammatory biomarkers

The levels of tumor necrosis factor-alpha (TNF-α) and interleukins (IL-6) in the serum were assessed using an ELISA kits from Quantikine Co., Minneapolis, MN, USA, following the manufacturer’s instructions. Immunoglobulin M (IgM) and immunoglobulin G (IgG) levels were measured in the serum using an ELISA kits from Cobas Company, USA, following the manufacturer’s instructions.

### Histological examination

Histological examination of the liver tissues was conducted following fixation using a 10% neutral buffered formalin solution. The tissues were dehydrated using ascending grades of ethyl alcohol (50–100%), then cleared in xylol (2/change ), then embedded in melted paraffin wax (60 c), blocked and sectioned with a thickness of 5 μm, and stained using the hematoxylin-eosin (H&E) staining method. The stained slides were examined under a light microscope for further analysis (Bancroft et al. 2013).

### Hepatic immunohistochemical analysis

The immunohistochemical staining of hepatic sections was carried out following the protocol described by Elshopakey and Elazab. in Elshopakey et al. 2021. Briefly, the hepatic sections were deparaffinized and rehydrated through a series of graded alcohol washes. For the sections prepared for anti-Cox-II, antigen retrieval was performed using 10-mM citrate buffer (pH 6.0) for 10–20 min, followed by a 20-min preservation step at room temperature and rinsing with distilled water. However, no antigen retrieval method was applied to the sections for caspase-3. To inactivate endogenous peroxidase, the sections were treated with 3% H_2_O_2_ in 100% methanol at 4 °C for 30 min and then washed with PBS. Subsequently, the slides were blocked with 10% normal blocking serum for 1 h at 25 °C. The primary antibodies, namely anti-caspase-3 (polyclonal rabbit anti-cleaved caspase-3 at dilution 1:100, BioCare Medical, Cat: CP229C, Concord, California, USA) and anti-Cox-II (monoclonal rabbit anti-Cox-II at dilution 1:100; ThermoFisher Scientific, Cat: RM-9121-S0, Fremont, CA, USA), were applied to the slides and allowed to incubate. Afterward, the slides were treated with biotinylated goat anti-rabbit IgG antiserum (Histofine kit, Nichirei Corporation, Tokyo, Japan) for 60 min and then washed with PBS. The final step involved adding streptavidin-peroxidase conjugate (Histofine kit, Nichirei Corporation) to the slides for a 30-min incubation period. The reaction was visualized by treating the slides with 3,3′-diaminobenzidine tetrahydrochloride (DAB)-H_2_O_2_ solution (pH 7.0) for 3 min. Following this, the slides were washed in distilled water and counterstained with hematoxylin.

### Statistical analysis

Statistical analysis was performed using SPSS version 22, and the data were presented as means ± standard error (SE). One-way ANOVA was employed for statistical analysis, and Duncan’s multiple range test with a 95% confidence limit (*p* < 0.05) was used to determine differences in means among the various samples **(**Snedecor and Cochran 1986**)**.

## Results

### Bioactive components in papaya seeds

Table 1 provides the quantification of antioxidant activity, total phenols, and total flavonoids in papaya seeds. The total phenolic and flavonoid contents in papaya seeds were found to be notably high, with 45.78 ± 0.81 mg GAE/g dry sample of seeds for total phenolic and 11.54 ± 0.23 mg QE/g dry sample of seeds for total flavonoids (Table 1). Furthermore, the antioxidant activity of papaya seeds was found to be significantly high, as determined by the DPPH free radical scavenging assay, highlighting their potential as a natural source of antioxidants.

**Table 1:** Bioactive components in papaya seeds

Parameters	Papaya seeds (PS)
Total phenolic (mg GAE/g dry sample)	45.78 ± 0.81
Total flavonoid (mg QE/g dry sample)	11.54 ± 0.23
DPPH (IC_50_ μg/mL)	87.56 ± 1.03

*GAE* gallic acid equivalent, *QE* quercetin equivalent

### HPLC analysis of papaya seeds

**Fig. 1:**
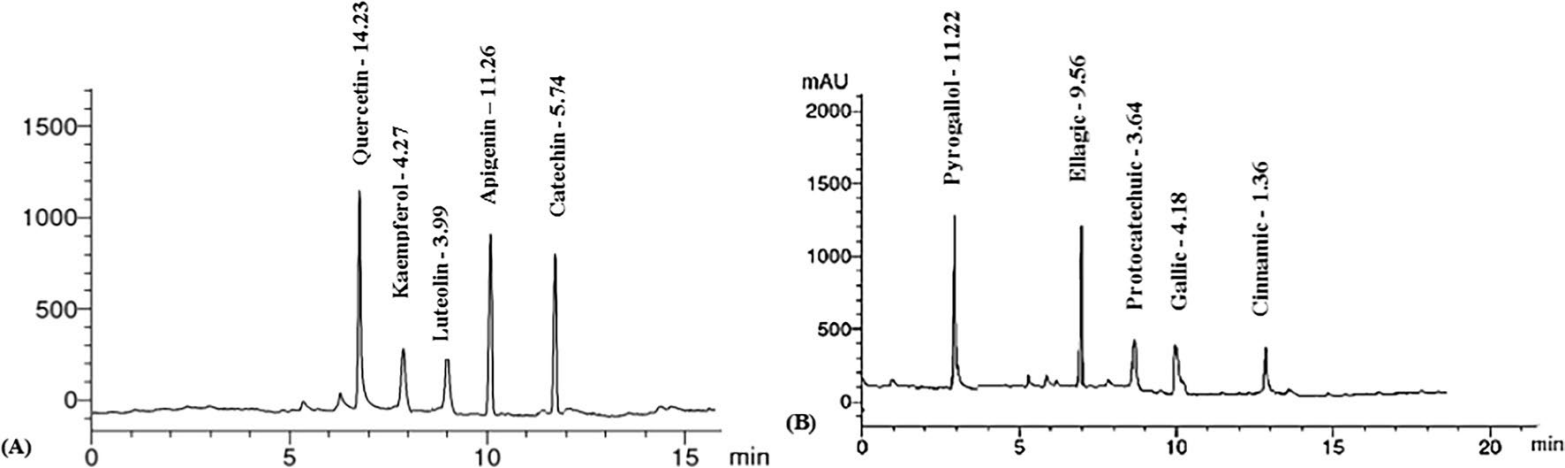
HPLC analysis of papaya seeds

HPLC analysis of papaya seeds resulted in the identification and quantification of 10 polyphenol compounds, including 5 flavonoids and 5 phenolic acids, as shown in Table 2. Among the flavonoids, quercetin, apigenin, and catechin were found to be the major compounds, present at concentrations of 14.23, 11.26, and 5.74 μg/mg, respectively. Regarding phenolic acids, pyrogallol, ellagic, and gallic acid were the predominant components identified in papaya seeds, with concentrations of 11.22, 9.56, and 4.18 μg/mg, respectively (Fig. 1 B; Table 2).

**Table 2:** HPLC analysis of papaya seeds

Components	RT (min)	Conc. (μg/mg)
Flavonoid compounds		
Quercetin	7.0	14.23
Kaempferol	7.9	4.27
Luteolin	9.1	3.99
Apigenin	10.0	11.26
Catechin	12.0	5.74
Phenolic compounds		
Pyrogallol	3.0	11.22
Ellagic	7.0	9.56
Protocatechuic	8.7	3.64
Gallic	10.0	4.18
Cinnamic	13.0	1.36

### Effects of cake enhanced with papaya seeds alone or combined with silymarin on blood count

The CCl4-exposed group of rats showed significant reductions in red blood cell count (RBCs), packed cell volume (PCV), and hemoglobin (Hb) compared to the control rats (*p* < 0.05) (Table 3). Treatment with cake enhanced with papaya seeds seemed to counteract the toxic effects of CCl4 on erythrocytes, potentially reducing the hemolytic effects induced by CCl4 administration (*p* < 0.05), as shown in Table 3.

**Table 3:** Effects of cake enhanced with papaya seeds alone or combined with silymarin on blood count

	Control	CPS	CCl4	CCl4+ S	CCl4 + CPS	CCl4 + CPS + S
RBCs (106/μl)	8.16 ± 0.24	8.51 ± 0.19	4.21 ± 0.30^a^	6.08 ± 0.36^ab^	5.89 ± 0.34^ab^	7.34 ± 0.14^b^
Hb (gm/dl)	14.07 ± 0.81	14.78 ± 0.83	6.64 ± 0.78^a^	11.21 ± 0.59^ab^	10.98 ± 0.68^ab^	12.73 ± 0.59^b^
PCV (%)	46.10 ± 1.52	47.65 ± 2.25	26.78 ± 1.57^a^	37.84 ± 1.82^b^	38.96 ± 1.27^ab^	43.16 ± 1.81^b^
WBCs (103/μl)	7.12 ± 0.87	7.08 ± 0.86	15.16 ± 1.52	9.59 ± 0.90^b^	10.61 ± 0.68^ab^	08.09 ± 0.97^b^
Lymphocyte (103/μl)	5.32 ± 1.05	5.09 ± 0.75	09.78 ± 1.06^a^	7.43 ± 0.65^ab^	7.11 ± 0.81^b^	6.98 ± 0.69^b^
Neutrophil (103/μl)	1.18 ± 0.52	1.09 ± 0. 32 ^a^	5.13 ± 0.37 ^a^	3.13 ± 0.31^ab^	4.73 ± 0.53^ab^	2.32 ± 0.29^ab^
Eosinophil ×103/μl	0.22 ± 0.02	0.21 ± 0.03 ^a^	0.16 ± 0.01^a^	0.19 ± 0.03^b^	0.20 ± 0.01^b^	0.21 ± 0.04^b^
Monocyte ×103/μl	0.24 ± 0.02	0.22 ± 0.02	0.36 ± 0.03^a^	0.27± 0.03^b^	0.28 ± 0.04^b^	0.26 ± 0.05^b^

Values expressed as mean ± SEM (*n* = 8). Significant difference versus ^a^ with respect to control and ^b^ with respect to CCl4

*CCl4* carbon tetrachloride, *S* silymarin, *CPS* cakes enhanced with papaya seeds

As depicted in Table 3, the CCl4-exposed group exhibited a significant decrease in WBCs, lymphocytes, and neutrophils, along with an elevation in toxic neutrophil numbers. However, treating CCl4-intoxicated rats with cake enhanced with papaya seeds combined with silymarin significantly increased total leukocytes and lymphocyte count, likely due to the known anti-inflammatory impacts of this treatment.

### Effects of cakes enhanced with papaya seeds on biochemical markers of hepatic injury

Table 4 presents noteworthy results indicating a significant increase (*p* < 0.05) in serum AST, ALT, and ALP levels in the CCl4 group compared to the control group. Nevertheless, in the treated groups (CPS, S, and CPS + S), these parameters displayed a partial restoration towards normal values, with CPS, S, and their combination demonstrating efficacy in reducing the activities of all liver function enzymes when compared to the CCl4 group. Notably, the combination of CPS and silymarin exhibited greater efficiency in mitigating the activities of these enzymes when compared to the individual CPS and S treatment groups.

**Table 4:** Effects of cakes enhanced with papaya seeds on biochemical markers of hepatic injury

Parameters	Control	CPS	CCl4	CCl4 + S	CCl4 + CPS	CCl4 + CPS + S
ALT (U/l)	38.09 ± 1.21	36.13 ± 1.40	118.09 ± 6.99^a^	80.13 ± 5.50^ab^	93.09 ± 2.94^ab^	50.38 ± 1.95^ab^
AST (U/l)	57.81 ± 2.77	56.28 ± 2.09	145.76 ± 9.18^a^	119.01 ± 6.0^ab^	126.2 ± 4.39^ab^	82.09 ± 5.37^ab^
ALP (U/l)	64.60 ± 1.93	62.76 ± 1.79^a^	148.75 ± 6.16^a^	113.65 ± 2.59 ^ab^	124.73 ± 3.51^ab^	90.47 ± 2.77 ^ab^
Total protein (g/dl)	7.42 ± 0.23	7.57 ± 0.29^a^	3.07 ± 0.24^a^	4.61 ± 0.13^ab^	4.29 ± 0.18^ab^	6.02 ± 0.11^ab^
Albumin (g/dl)	4.31 ± 0.12	4.51 ± 0.09^a^	1.08 ± 0.11^a^	2.54 ± 0.24^ab^	2.87 ± 0.18^ab^	3.10 ± 0.14^a^
Globulin (g/dl)	3.11 ± 0.1	3.06± 0.2^a^	1.99 ± 0.13^a^	2.07 ± 0.12^ab^	1.42± 0.06^ab^	2.92 ± 0.32^ab^

Values expressed as mean ±SEM (*n* = 8). Significant difference versus ^a^ with respect to control and ^b^ with respect to CCl4

*CCl4* carbon tetrachloride, *S* silymarin, *CPS* cakes enhanced with papaya seeds

As depicted in Table 4, the CCl4 exposed group exhibited a significant reduction in serum albumin, total protein, and globulin levels compared to the control group (*p* < 0.05). However, the CPS group demonstrated a noteworthy increase in total protein, albumin, and globulin levels in contrast to the CCl4-exposed group (*p* < 0.05). Furthermore, the administration of cakes enriched with papaya seeds in combination with silymarin proved to be even more effective in elevating albumin, total protein, and globulin levels (*p* < 0.05), as indicated in Table 4.

### Effects of cakes enhanced with papaya seeds alone or combined with silymarin on antioxidant/oxidative stress parameters

As presented in Table 5, the hepatic levels of MDA and NO were significantly increased in rats treated with CCl4 compared to the control group (*p* < 0.05). Concurrently, the hepatic activity of SOD and GSH was significantly reduced in the CCl4 group when compared to the control rats (*p* < 0.05). However, the administration of cake enhanced with papaya seeds (CPS) effectively attenuated the oxidative stress induced by CCl4 intoxication, leading to a significant reduction in MDA and NO levels in comparison to the CCl4 group (*p* < 0.05), as displayed in Table 5. Furthermore, feeding with CPS significantly boosted the activity of SOD, GSH, and CAT levels compared to the control group (*p* < 0.05) (Table 5). Notably, CPS intake mitigated the decline in antioxidant biomarkers caused by CCl4 intoxication, as indicated in Table 5. Remarkably, the rats subjected to CCl4 intoxication and treated with cake enhanced with papaya seeds along with silymarin exhibited the most favorable outcomes in terms of antioxidant effects, surpassing the other groups (Table 5). These results suggest that CPS, especially in combination with silymarin, has the potential to offer substantial protection against oxidative stress induced by CCl4 and can effectively enhance antioxidant defense mechanisms in the liver.

**Table 5:** Effects of cakes enhanced with papaya seeds on hepatic oxidative stress parameters

Parameters	Control	CPS	CCl4	CCl4 + S	CCl4 + CPS	CCl 4+CPS + S
GSH (mg/g tissue)	6.67 ± 0.35	7.29 ± 0.41	1.65 ± 0.49^a^	4.35 ± 0.36^ab^	4.08 ± 0.53^ab^	5.63 ± 0.19^b^
NO (μmol/g tissue)	33.71 ± 2.89	30.87 ± 2.67	69.46 ± 2.65^a^	46.90 ± 2.19^ab^	53.10 ± 3.93^ab^	40.89 ± 2.23^b^
MDA (nmol/g tissue)	50.29 ± 3.06	47.11 ± 2.48	90.06 ± 3.22^a^	75.1 ± 2.37a^b^	64.00 ± 3.63^ab^	57.90 ± 3.75^b^
SOD (U/g tissue)	348.6 ± 13.87	366.9 ± 12.94	220.5 ± 09.90^a^	312.5 ± 12.96^b^	289.51 ± 09.53^ab^	322.4 ± 16.15^b^

Values expressed as mean ± SEM (*n* = 8). Significant difference versus ^a^ with respect to control and ^b^ with respect to CCl4

*CCl4* carbon tetrachloride, *S* silymarin, *CPS* cakes enhanced with papaya seeds

### Effects of cake enhanced with papaya seeds alone or combined with silymarin on pro-inflammatory cytokine biomarkers

The rise in serum TNF-α and IL-6 levels observed in the CCl4 group (*p* < 0.05) indicates liver injury, as demonstrated in Table 6. However, administering CPS to CCl4-intoxicated rats resulted in a significant reduction in TNF-α and IL-6 levels compared to the CCl4 group (*p* < 0.05) (Table 6). Furthermore, the combination of CPS with silymarin proved to be more effective than other groups (Table 6).

**Table 6:** Effects of CPS on inflammation and immunoglobulins biomarkers

	Control	CPS	CCl4	CCl4 + S	CCl4 + CPS	CCl4 + CPS + S
TNF-α (pg/ml)	51.21 ± 3.05	45.11 ± 4.09	171.75 ± 6.51	82.68 ± 4.16	90.10 ± 4.75	65.18 ± 4.67
IL-6 (pg/ml)	38.63 ± 1.67	30.43 ± 1.5	107.66 ± 4.99^a^	78.51 ± 3.34 ^ab^	80.40 ± 3.69 ^ab^	57.32 ± 3.80 ^b^
IgG (ng/ml)	6.53 ± 0.34	6.64 ± 0.39	2.71 ± 0.20 ^a^	3.52 ± 0.38 ^ab^	3.19 ± 0.46 ^ab^	5.39 ± 0.21^b^
IgM (ng/ml)	3.22 ± 0.24	3.41 ± 0.20	0.70 ± 0.06 ^a^	2.12 ± 0.21 ^ab^	1.86 ± 0.36 ^ab^	2.89 ± 0.42^b^

Values expressed as mean ± SEM (*n* = 8). Significant difference versus ^a^ with respect to control and ^b^ with respect to CCl4

*CCl4* carbon tetrachloride, *S* silymarin, *CPS* cakes enhanced with papaya seeds

The data presented in Table 6 demonstrates the impact of cake enhanced with papaya seeds on the serum immunoglobulin levels (IgG and IgM) in rats with induced immunotoxicity. Injection of CCl4 to induce immunotoxicity resulted in a significant decrease (*p* < 0.05) in the mean values of IgG and IgM compared to the control group (Table 6). However, feeding the rats with CPS significantly increased (*p* < 0.05) the levels of IgG and IgM compared to the CCl4 group (Table 6). Notably, there were significant differences (*p* < 0.05) in the levels of IgM and IgG between the CCl4-intoxicated rats treated with CPS only, S only, and CPS + S. The CPS + S group exhibited the highest levels of immunoglobulins IgG and IgM, as indicated in Table 6.

### Histopathological results

**Fig. 2:**
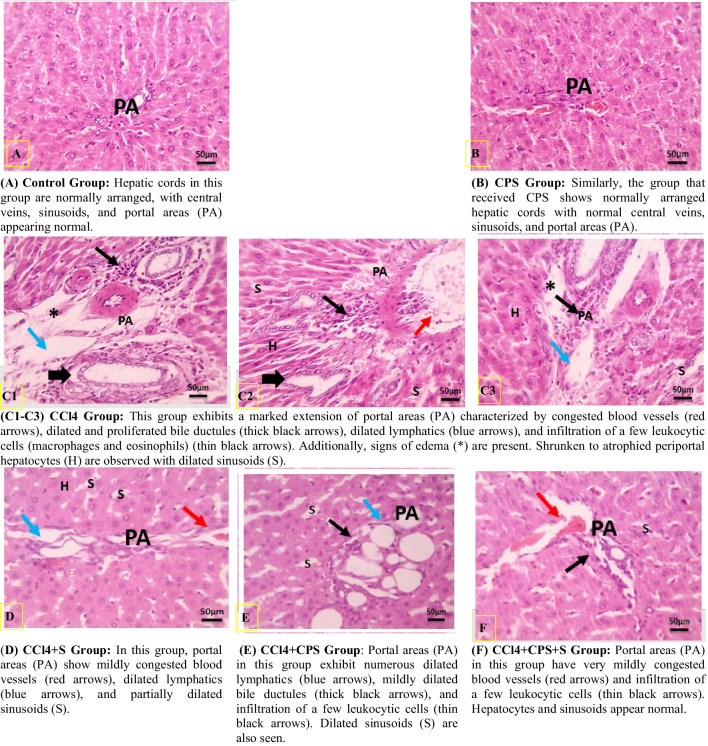
Microscopic pictures of hepatic sections (H & E, 400× magnifications, scale bar: 50 μm)

Histological examination of liver sections revealed distinct findings among the experimental groups. In Fig. 2A (control group) and B (CPS-treated group), hepatic sections showed normal hepatic structure with regular hepatic cords and central veins. In contrast, Fig. C1, C2, and C3 in the CCl4 group displayed hepatic abnormalities, including increased portal area thickness, the presence of inflammatory cells, dilated bile ductules, congested blood vessels, and dilated lymphatics. Additionally, there was evidence of edema (Fig. C2) and shrunken periportal hepatocytes with dilated sinusoids (Fig. C3). On the other hand, the CCl4 + S group had mildly congested portal area blood vessels, along with dilated lymphatics and partially dilated sinusoids (Fig. 2D). Meanwhile, the CCl4 + CPS group exhibited livers with numerous dilated portal area lymphatics, mildly dilated bile ductules, and limited leukocytic cell infiltration (Fig. 2E).Remarkably, the CCl4 + CPS + S group displayed portal areas with minimal blood vessel congestion and leukocytic cell influx, closely resembling the control group’s structure (Fig. 2F).

### Immunohistochemical results

**Fig. 3:**
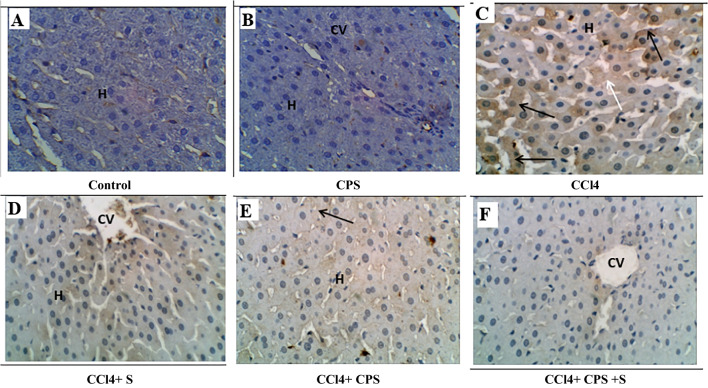
Immunohistochemical staining in liver sections different experimental groups by caspase-3. Brown color indicates immunopositivity for each stain, CV, central vein. H, hepatocyte. Hepato-portal area, HP. Magnification: 40×

**Fig. 4:**
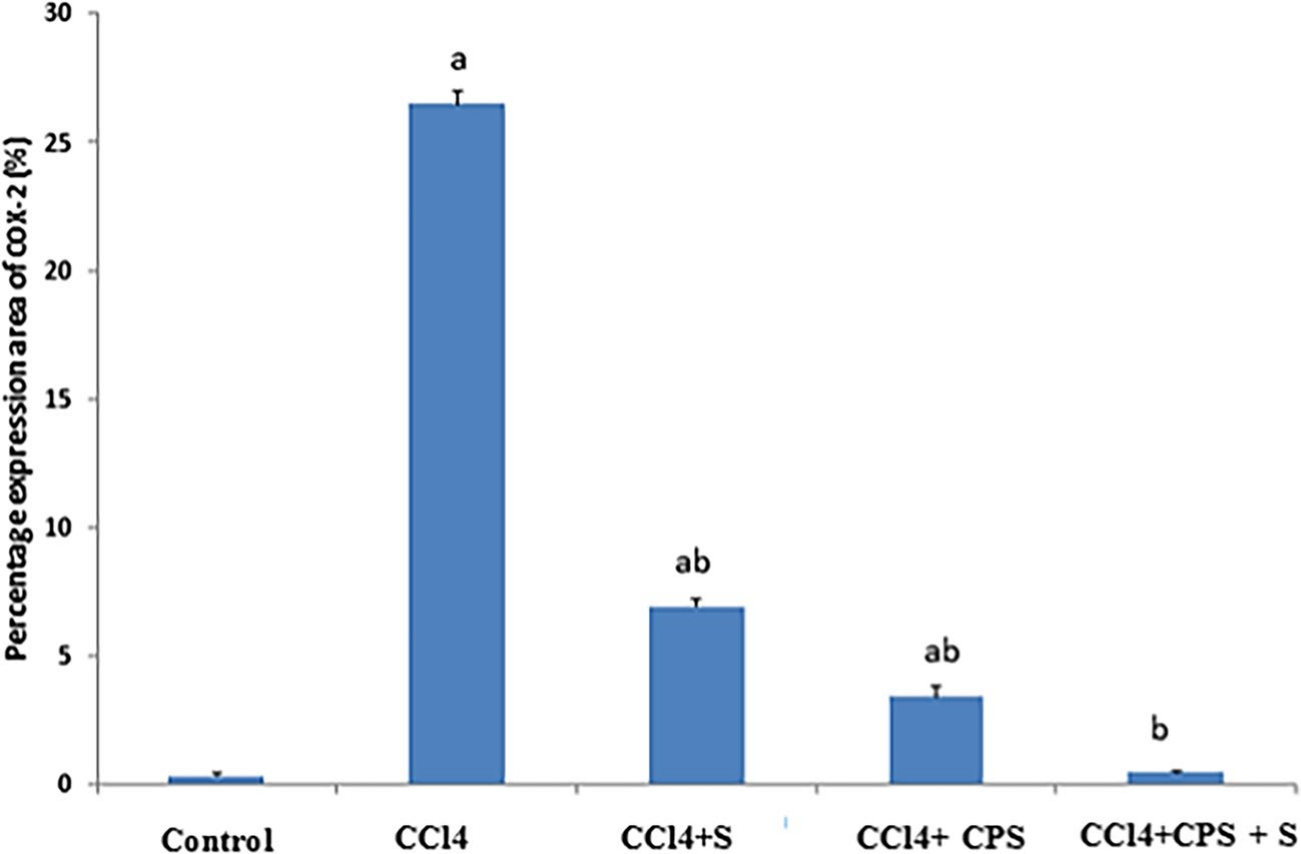
Percentage expression area of caspase-3 between different treatment groups. Significant difference versus ^a^ with respect to control and ^b^ with respect to CCl4

Figure 3A, B, C, D, E, and F and Fig. 4 demonstrate alterations in caspase-3 expression in hepatic tissues. In the normal group, caspase-3-immunolabeled cells were rarely present in the liver of control rats (Fig. 3A). In the CPS group, there was no expression of caspase-3 immunostain (Fig. 3B). In the CCl4 group, brown staining was observed, indicating caspase-3 immunolabeled hepatocytes (black arrows), suggesting an inflammatory response and a slight increase in apoptotic cells (white arrow) (Fig. 3C). In the CCl4 + S group, moderate caspase-3-immunolabeled hepatocytes were present around the irregular central vein (Fig. 3D). In the CCl4 + CPS group, there was a slight decrease in caspase-3-immunolabeled cells (black arrow), indicating a slight improvement in the liver section (Fig. 3E). In the CCl4 + S+ CPS group, a decrease in caspase-3-immunolabeled cells was noticed, indicating that hepatocytes were nearly normal compared to the control group (Fig. 3F). Therefore, it is highly possible that feeding with CPS or feeding with CPS along with silymarin can prevent the dysregulation of caspase-3 induced by CCl4.

**Fig. 5:**
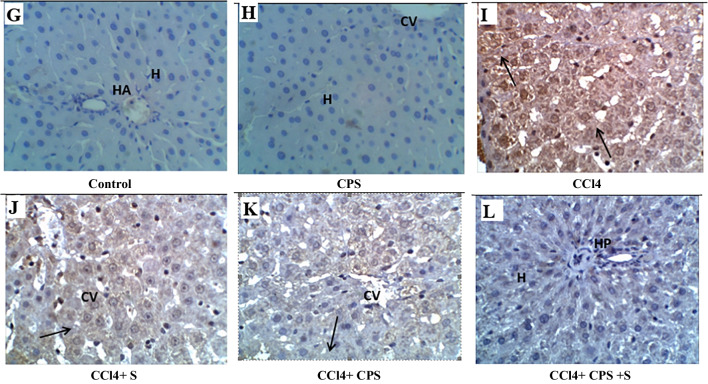
Immunohistochemical staining in liver sections different experimental groups by COX-2 (E:H) immunostaining. Brown color indicates immunopositivity for each stain, CV, central vein. H, hepatocyte. Hepato-portal area, HP. Magnification: 40×

**Fig. 6:**
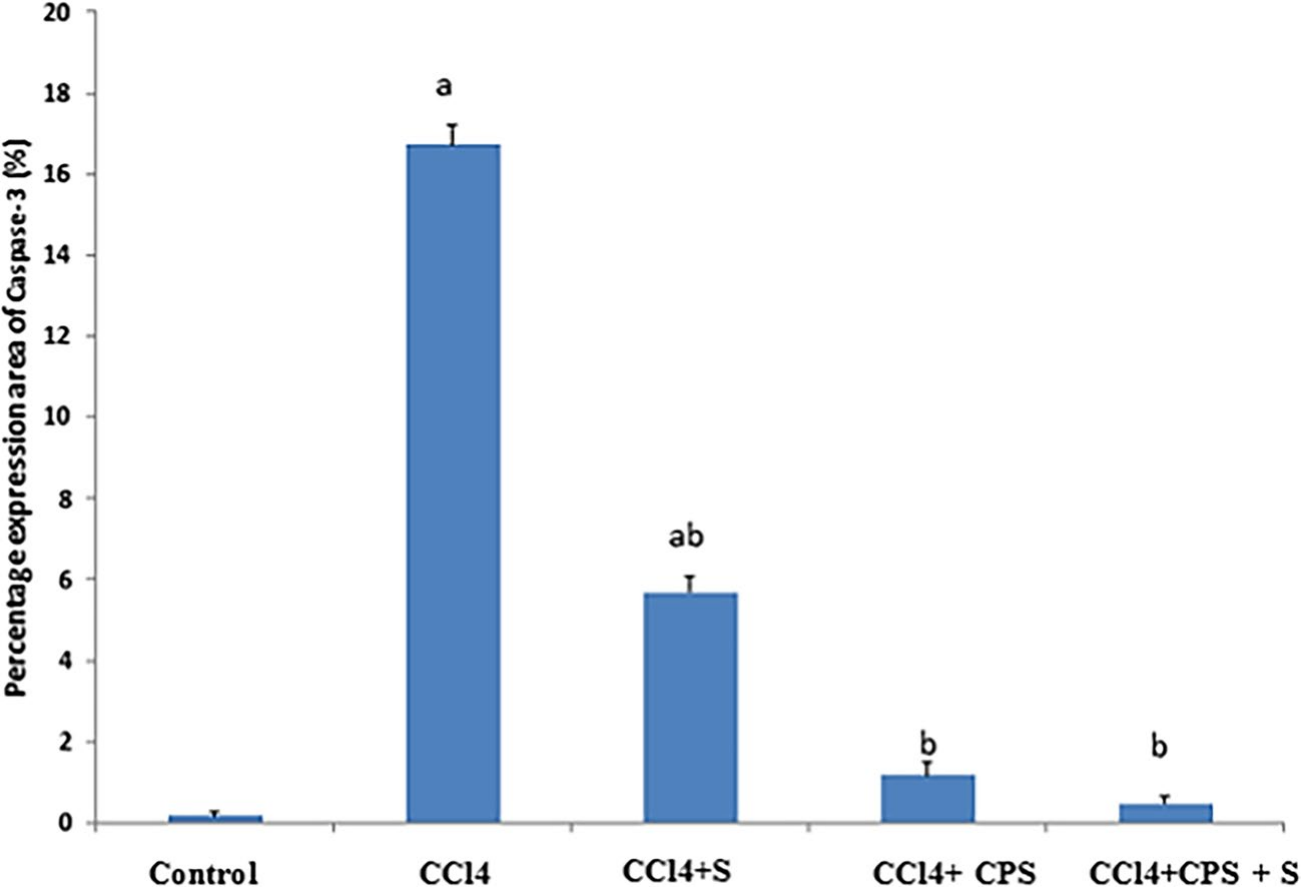
Percentage expression area of COX-2 between different treatment groups. Significant difference versus a with respect to control and b with respect to CCl4.

Figure 5G, H, I, J, K, and L and Fig. 6 show COX-2 expression and changes in hepatic specimens. In the normal group (Fig. 5G), there is negligible COX-2 immunostain in hepatic cells around the normal hepatic portal area (HA). Similarly, in the CPS group (Fig. 5H), there is no expression of COX-2 immunostain in hepatic cells around the normal central vein (CV). In the CCl4 group (Fig. 5I), there is a strong increase in cytoplasmic staining of COX-2 (arrows) in apoptotic hepatocytes, indicating hepatic injury. In the CCl4 + S group (Fig. 5J), only a few hepatocytes and inflammatory cells show positive staining for COX-2. In the CCl4 + CPS group (Fig. 5K), there is a reduced expression of cleaved COX-2 (arrow) in hepatocytes, indicating moderate cellular amelioration. Finally, in the CCl4 + S + CPS group (Fig. 5L), only a few cells show positive expression for cleaved COX-2, resembling the nearly normal control group in the hepato-portal area. The results indicate that feeding with CPS alongside silymarin was superior compared to the other treatment groups, namely CCl4 + S or CCl4 + CPS.

## Discussion

The nutritional value of food is often assessed by its total flavonoid and phenolic content, as these compounds serve as indicators of the medicinal properties of natural products (Zakia et al. 2015). The findings suggest that papaya seeds are abundant in flavonoids and phenolic compounds, contributing to their potential medicinal properties. The presence of various polyphenol compounds in papaya seeds (Table 2 and Fig. 1) highlights the medicinal significance of papaya as a plant with potential health benefits. One specific compound found in papaya seeds, ellagic acid, has been shown to exhibit protective effects by promoting the healing of intestinal damage and reducing the expression of pro-inflammatory factors in the intestine (Sun et al. 2017). This suggests that papaya seeds, particularly ellagic acid, could be beneficial for gastrointestinal health and may have potential applications in treating gastrointestinal ailments.

The CCl4-exposed group exhibited significant reductions in RBCs, PCV, and Hb. These reductions are likely due to disrupted hematopoiesis and erythrocyte damage. CCl4-induced macrocytic hypochromic anemia could be linked to several factors, including membrane protein degradation, increased lipid peroxidation, and altered membrane-bound enzymes (Makni et al. 2012). Elevated toxic neutrophil counts observed in this group may result from the release of free radicals during CCl4 metabolism, impacting the integrity and structure of white blood cells (Sinha et al. 2006). Treatment with papaya seed-enhanced cake appeared to mitigate erythrocyte toxicity and decrease leukocyte and lymphocyte counts. This effect may be attributed to its anti-inflammatory properties, particularly when combined with silymarin.

Liver enzymes, specifically ALT, AST, and ALP, have been widely recognized as reliable indicators of CCl4-induced hepatotoxicity, as established by numerous researchers (Gazwi and Magda 2019). In response to CCl4 exposure, the liver undergoes substantial structural damage, resulting in a significant elevation of these biochemical markers for liver injury. This increase is attributed to the release of these enzymes from the cytoplasm into the bloodstream following cellular and mitochondrial destruction (Mir et al. 2010). Consistent with previous investigations (Ahmed et al. 2021), our study also observed a significant increase in hepatic marker enzymes in CCl4-intoxicated mice, confirming prior findings. According to our data, the administration of silymarin in combination with cakes enriched with papaya seeds showed superior results compared to using cake alone or silymarin alone.

In the case of CCl4-intoxicated rats, a substantial and notable decrease in serum total protein and albumin levels was observed. This decline is likely attributed to impaired liver function and damage to hepatic cells caused by CCl4 exposure (El Rabey et al. 2021). However, in the case of CCl4-intoxicated rats treated with cake enhanced with papaya seeds combined with silymarin, a significant increase in protein levels was observed, surpassing the effects of the groups treated with cake enhanced with papaya seeds alone or silymarin alone. This improvement can be attributed to the potent antioxidant and protective properties exhibited by both papaya seeds and silymarin. The combined action of these two components appears to have a more substantial positive impact on protein levels, leading to better results compared to using either papaya seeds or silymarin alone.

Carbon tetrachloride (CCl4) is commonly used to induce hepatic oxidative damage and fibrosis in animal models due to its ability to closely mimic the effects observed in humans. As a result, CCl4 serves as a valuable tool for evaluating the hepatoprotective properties of various drugs (Domitrović and Jakovac 2011).

CCl4-induced hepatic fibrosis is closely associated with an exacerbation of lipid peroxidation and a reduction in antioxidant defenses. This occurs through the generation of highly reactive trichloromethyl-peroxyl radicals (−OOCCl3) during the metabolism of CCl4. These radicals initiate the process of lipid peroxidation, leading to the production of by-products such as MDA, which disrupts the integrity of cellular membranes and can ultimately cause cell death (Weber et al. 2003). Additionally, trichloromethyl (−CCl3) radicals are formed, and they can react with reduced compounds like GSH, resulting in its depletion (Boll et al. 2001). In the current study, the liver oxidative stress parameters, including MDA, GSH, SOD, and NOx, were analyzed. The results showed a tendency for reduced levels of GSH and SOD, while MDA and NOx levels exhibited an increasing trend in the livers of CCl4-intoxicated mice. These findings align with other studies that have reported similar effects induced by CCl4 (Ahmed et al. 2021). Moreover, CCl4 intoxication significantly depleted hepatic SOD, GSH, and GSH levels due to the excessive production of free radicals (Elshopakey et al. 2021).

The group of rats treated with cakes enhanced with papaya seeds demonstrated a significant reduction in MDA and NOx concentrations, coupled with a notable elevation of GSH and SOD levels. These observations align with the protective, antioxidant, and free radical scavenging properties attributed to papaya seeds in countering liver injury induced by CCl4. The antioxidants present in papaya seeds play a crucial role in the detoxification of various toxic chemicals and xenobiotics (Olakunle 2010).

Papaya seeds are rich in natural antioxidants, including quercetin, kaempferol, luteolin, apigenin, catechin, pyrogallol, protocatechuic,and gallic acid, all of which contribute to their promising antioxidant properties. Notably, CPS’s superior antioxidant activity is attributed to the presence of several phenolic components, such as ellagic acid, punicalin, and punicalagin, rather than relying on a single pure polyphenol (as evident from Table 2). Gallic acid and ellagic acid, two potent free radical scavengers found in papaya seeds, play essential roles in restoring hepatic enzyme activity, including peroxidase, catalase, and SOD, thereby supporting the liver’s antioxidant defense system. Additionally, they help suppress lipid peroxidation, which further contributes to the overall protective effects against liver damage induced by CCl4 (Ashoush et al. 2013). In summary, the inclusion of papaya seeds in the cakes led to a substantial improvement in antioxidant status, contributing to their beneficial effects in mitigating CCl4-mediated liver injury through their potent free radical scavenging and antioxidant properties. One intriguing finding from this research is that the combination of CPS (papaya seed extract) with silymarin demonstrated a shielding impact against oxidative stress and positively influenced the antioxidant redox system.

CCl4 exposure was found to activate NF-κB, leading to increased production of pro-inflammatory cytokines, such as TNF-α and IL-1β, recognized as major mediators of hepatotoxicity (Geier et al. 2002). The current experiment’s findings align with those of Elshopakey et al. (2021), who demonstrated that CCl4 administration elevated serum TNF-α and IL-6 levels in rats. Notably, the consumption of cake enriched with papaya seeds significantly decreased TNF-α and IL-6 levels, indicating its potential anti-inflammatory effects. Nonetheless, the combined administration of CPS and silymarin led to the restoration of inflammatory markers, approaching the levels observed in the normal control group. This effect could be attributed to the anti-inflammatory and immunomodulatory properties of these substances, which appear to offer beneficial actions in mitigating inflammation.

The reduction in immunoglobulins caused by CCl4 injection is consistent with the findings of Bystry et al. (2011), who reported that CCl4 acts as a chemoattractant for natural killer cells, monocytes, and various other immune cells, leading to immunotoxic effects.

The inclusion of cake enriched with papaya seeds demonstrated a notable improvement in the concentrations of IgM and IgG in rats with induced liver cirrhosis. This enhancement of immune functions can be attributed to the chemical composition of papaya seeds, which contain high levels of antioxidant compounds, such as phenolics and flavonoids. Antioxidants play a crucial role in reducing DNA damage, limiting lipid peroxidation, maintaining proper immune system function, and inhibiting cancerous modifications in vitro, thus potentially preventing certain diseases.

Polyphenols, including phenolic acids and bio-flavonoids, are the major constituents of papaya seeds. Studies have shown that polyphenols from various sources have a modulatory effect on epigenetic mechanisms, such as genetic methylation, histone modifications, and post-transcriptional regulation by microRNAs. These mechanisms, in turn, can influence the immune system, affecting both the activation and differentiation of multiple cellular types involved in the immune response (Tsao 2010). By providing a rich source of polyphenols and antioxidants, papaya seeds present promising potential in bolstering immune functions and maintaining overall health. This suggests that incorporating papaya seeds into the diet through the consumption of cake enhanced with these seeds may be beneficial in supporting the immune system and reducing the risk of certain diseases (Cuevas et al. 2013).

CCl4 intoxication led to a significant increase in collagen deposition, particularly in the portal areas, along with noticeable degeneration and disruption of hepatocyte structure. These results are consistent with findings reported by Al-Rasheed et al. in Al-Rasheed et al. 2015. However, when treated with cakes enriched with papaya seeds, a decrease in collagen deposition and an improvement in regenerative hepatocytes were observed. The normal architecture of hepatocyte cords was only slightly affected, with only a few areas of discontinuity noticed. Notably, in the group treated with cakes enhanced with papaya seeds combined with silymarin, there was a marked improvement in liver architecture, evident hepatocyte regeneration, and minimal degenerative changes.

The research outcomes unequivocally demonstrated that CCl4 induced a substantial elevation in the immunohistochemical overexpression of pro-inflammatory mediator COX-2, compared to the results observed in the normal group. These findings align with the results reported in other similar studies by Popović et al. (2019) and Dong et al. (2019). Importantly, our study strongly emphasizes that the administration of CPS accompanied by silymarin effectively safeguards the liver against CCl4-induced damage. This protective effect can be attributed, at least in part, to the potent antioxidant and anti-fibrotic actions of CPS.

Lipid peroxidation and free radicals generated during CCl4 poisoning lead to mitochondrial DNA depletion and damage, as well as alterations in the cell’s structure. This, in turn, results in a modification of the mitochondrial membrane potential (Knockaert et al. 2012). The considerable reduction in mitochondrial membrane potential indicates a severe impairment of membrane permeability and integrity (Knockaert et al. 2012). Consequently, mitochondrial permeabilization and dysfunction trigger the release of proapoptotic proteins, ultimately causing cellular apoptosis and necrosis (Tian et al. 2019). Caspase-3, a crucial member of the apoptotic protease family, plays a significant role in cellular apoptosis, with its level serving as an indicator of the extent of apoptosis (Hu et al. 2014). The findings from the study revealed that the co-administration of CPS and silymarin could effectively suppress the elevation of caspase-3 levels induced by CCl4, suggesting a protective effect on mitochondria and an inhibitory impact on apoptosis and necrosis. The obtained result is consistent with the findings of Patel et al. (2010), wherein they reported that silymarin demonstrated a reduction in apoptotic cell death related to hepatotoxicity.

## Conclusion

In conclusion, cakes enhanced with papaya seeds exhibit immunostimulant effects and protect against CCl4-induced immunotoxicity and hepatotoxicity in rats. Papaya seeds contain valuable phytochemicals with antioxidant properties. The cakes improved hematological parameters, reduced hepatotoxicity, enhanced antioxidant defenses, and restored immune markers. Histopathological analysis revealed amelioration of hepatocellular necrosis and reduced caspase-3 expression, indicating hepatocellular protection. Combining papaya seeds with silymarin enhanced the protective effects. These findings suggest that papaya seed-enhanced cakes have potential as functional ingredients to support immune health and combat liver damage.

## Data Availability

The datasets utilized and analyzed during this investigation are available upon reasonable request from the corresponding author.
